# Use of Call Centers in Polio Eradication Efforts in Island Settlement in Chad

**DOI:** 10.29245/2578-3009/2021/S2.1113

**Published:** 2021-04-15

**Authors:** Adele Daleke Lisi Aluma, Sam Koulmini, Souley Kalilou, Obianuju Igweonu, Amadou Felix Kouassi, Mohamed Alimou Traore, Benoit Ntezayabo, Laurel Zomahoun Delayo, Aboubacar Barry, Aime Matela Esanga, Adama Nanko Bagayoko, Don Jethro Mavungu Landu, Abdel Aziz Kadai, Bondoro Toyma, Djibrine Abakar Sedick, Penaling Nathei, Daouda Mahamat, Philbert Bohoussou, Joseph Okeibunor, Narcisse de Medeiros, Bakoly Rabenarivo, Fabien Dio-mande, Sam Okiror

**Affiliations:** 1 Independent Consultant; 2 Ministry of Health Chad; 3 University of Nigeria, Nsukka; 4 WHO Chad; 5 WHO Headquarters, Geneva; 6 WHO Regional Office for Africa (WHO AFRO), Brazzaville, Congo; 7 UNICEF, Dakar; 8 WHO Horn of Africa Coordination Office (HOA), Nairobi KENYA

**Keywords:** Polio, Call Centers, Immuninzation, Chad

## Abstract

**Background:**

One of the four key strategies of the Global Polio Eradication Initiative (GPEI) is high immunization coverage, with oral polio vaccine as part of routine immunization schedules. However, given the weak routine immunization structures in the African Region, coverage is enhanced with supplemental immunization activities (SIAs), and mop-up immunizations. Unfortunately, anecdotal information show that vaccination teams sometimes omit some catchments areas without immunization. This paper thus describes the use of *“Call Centers”* in detecting missed populations and taking prompt corrective action.

**Method:**

The study was based on review of call records during polio supplemental immunization campaigns in Bol Districts in Chad from February to May 2018. The immunization coverage resulting from these campaigns was compared with that of February 2018. A compilation of data – details on communities, community leaders, and their phone numbers was performed. On the eve of the campaign, community leaders were alerted on the vaccinators’ visitThe community leaders were called on the eve of the campaign to alert them on the visit of the vaccinators. At the end of each day, activities (visits as well) were reviewed at the coordination centres Vaccinators were asked to return to any community where community leaders did not confirm visits).

**Result:**

Telephone calls allowed the verification and confirmation of the vaccinators visits in 92% of cases. Villages where vaccination was planned but which were not reached were revisited. More than 1,011 children were caught up through this approach in 10 villages in the Bol district.

**Conclusion:**

In conclusion, call centers played significantly higher role in generating covering more children with immunization during immunization campaign.

## Introduction

The recent successes in polio eradication globally and in the World Health Organization, African Region, are evident. Cases of wild poliovirus (WPV) types 2 and 3 (WPV2 and WPV3) were last reported in 1999 and 2012 respectively^1^. Cases of poliovirus Response to Polio Virus Disease Outbreaks in the Horn of Africa and Lake Chad Basin type 1 (WPV1), declined by almost fourteen-fold (59 to 4) between 2013 and 2016^2,3^. The Region was course of chalking two years with no case of wild poliovirus since the last case reported on 24^th^ July 2014 in Nigeria, until four new cases were reported in the same area in Borno state, between July and August 2016.

This new outbreak, once again, returned the countries in the Lake Chad sub-region, including Chad, in a list of countries at risk for poliovirus transmission. The necessary response saw the declaration of this outbreak to be a regional public health emergency and is implementing a regional outbreak response, coordinated with neighbouring countries. The four key strategies of the Global Polio Eradication Initiative (GPEI) namely, high coverage with routine oral polio vaccine in immunization schedules, surveillance for acute flaccid paralysis, supplemental immunization activities (SIAs), and mop-up immunizations, were activated in responding to the Nigerian outbreak within the lake Chad sub-region, including the Republic of Chad. The sub region, and indeed Chad remained at risk for wild poliovirus (WPV) because of several factors mainly attributable to poor quality and immunization coverage^4-7^.

The Lake District has two major challenges, namely, supplying vaccines to the island sub-districts, and reaching out to the remote villages with power difficult access, characterized by a lack of qualified human resources, during the routine immunizations programmes and even during the Supplementary Immunizations Activities “SIA”. In Lake Chad Basin’s Contingency Plan for Polio Response, planning established specific strategies to improve immunization activities in Island areas. These particularly focused on market places, the nomads, refugees and IDPs, where children 0-10 years were found with low immunity, including a significant number of zerodose or never-vaccinated children and partially or totally uncovered villages during the routine immunization, intensive vaccination activities (IVA) and supplementary immunization activities (SIAs).

During our first interventions in the Bol Islands, it was observed that vaccination was only conducted in routine and static approaches. In addition, advanced immunization strategies were almost non-existent. Approximately 90% of children vaccinated in our first intervention had never received any other vaccine including that against Polio. Villages were found where all children aged 0-5 years had never been vaccinated.

To tackle this major challenge, the district task force, the WHO support team / Task Team and UNICEF’s Bol district-based team put in place an experimental strategy during the April 2018 Polio SIA. This strategy consisted on informing all island villages leaders, the camp and Canton ferrick’s in Bol district, on a regular and systematic basis, to ensure that they were informed (on the eve) of vaccinators visits and their subsequent confirmation or denial on the actual visits at the end of the day. This activity of tracking the vaccinators movements through the local and traditional leaders was called “Call center”.

Such passive surveillance strategy has been used for Ebola in Guinea, principally with a telephone alert system^8^. Community members and health facilities report deaths and suspected Ebola cases to local alert numbers operated by prefecture health departments or to a national toll-free call center. The national call center additionally functions as a source of public health information by providing answers to the public on Ebola^8^.

This innovative strategy, which was piloted in the Banangorea area of responsibility during the April SIA, proved to be effective. For the first time the district executive team was able to achieve a daily assessment of the coverage of the villages, nomadic ferricks and camp of fishers, thanks to the consistent and continuous communication with the leaders of the different local stakeholders involved. Following its success, the strategy was extended to cover all the activities of boosting immunity of the populations of island areas, ferricks, and nomadic camps throughout Bol health district. This paper documents the experiences and lessons learnt in using this strategy to boost immunization in the Island settlements of Chad.

## Methods

### Study area setting

We conducted an action-research study involving 12 sub-districts of Bol (Chad): Banangorea, Fitine, Ngarangou, Matafo, Urban Bol, Kinaserom, Melea, Kangallam, Bougourmi, Tchongolet, Merom and Sawa where an innovative approach was tested. The study was conducted in three phases, from February 1 to May 31, 2018, with 3 rounds of vaccination campaign for children aged 0-10 years. The first phase concerned the development of innovative strategies called “call centers for polio efforts” aimed to improve the vaccination coverage for the district of Bol. The second phase consisted of the experimentation of this approach in a sub district. In the third phase, a benchmarking was carried out based on the first experiment, which allowed extending the approach throughout the district of Bol. This period was marked by security instability in the Lake chad islands following operations of the terrorist group Boko Haram. The various attacks of the latter had a negative impact on health activities in the region.

### Vaccination activity

#### Targets

children under 10 years old.

#### Vaccination stakeholders

WHO, UNICEF, management team of the district, the team of the sub-district, vaccinators, the pointers, outreach supervisors, main supervisor.

#### Usual preparation for vaccination

Before vaccination activity, a microplanning update was first carried out. The managers of Sub-district, vaccinators and social mobilisers were briefed on the polio situation, route and dosage of the vaccine, the messages to be conveyed to parents and caregivers, number of teams per area of responsibility and the budget. Finally, the vaccination progress plan for each health area was presented and validated.

#### Usual vaccination schedule

The actual vaccination took place over three days and one catch-up day. The vaccine was stored in Sub-districts, which each morning provided the required daily doses to each vaccination team. For remote or hard-to-reach areas, vaccination teams received the doses for all vaccination days at once. After vaccination, the vaccinators brought the tally sheets back to Sub-districts. The health centres compiled these tally sheets and sent them to the district level. The results sent to the district were compared with the results of telephone calls from village, ferry and fishing camp chiefs. An evaluation was carried out at the end of each day. This daily evaluation helped to resolve some of the difficulties encountered during the vaccination. Corrective actions were decided during this meeting.

After the vaccination campaign, partners and the district management team evaluated the campaign. This evaluation focused on the number of villages reached. Afterwards, partners, the district management team, managers of health facilities and EPI managers held feedback meetings, particularly on activities according to the framework established by the district management team and the call centre activity organised by partners. Finally, they wrote the campaign report.

### Verification of the effectiveness of vaccination in the villages

To confirm whether vaccinators had reached all the targets (villages and children under 10 years of age), a telephone verification system was set up and implemented.

**a)Call centre for polio control efforts**

The main tool in the call centre approach to polio control efforts was the telephone.

**b) Description of the polio call centre approach**

The implementation of the call centre approach was carried out in three phases:

### Phase 1 and 2: Design of the call centre approach and conduct of the pilot project

A first meeting was organised between the Task Team consultant (lead coordinator) and the District Management Team. This meeting made it possible to design the call centre approach and to select the BANANGORE sub-district as the first site for testing the approach during the first campaign in February 2018.

A second meeting, named a “community meeting”, was organised by the consultant with various stakeholders in BANANGORE village, including the village chiefs, the representative of district chief, the team of Sub-districts, community workers and members of the health committee. The purpose of this meeting was to raise community awareness of the vaccination campaign, validate the vaccinators’ progress plan and develop the telephone directory to include the village chiefs. A copy of the directory and vaccinators’ progression plan was given to the Task Team consultant and another to the canton chief representative.

### Phase 3: Comparative evaluation of the call centre approach in BOL district

A first meeting was organised by the District Executive Team with the support of partners (WHO, UNICEF, Task Team). The objective of this meeting was to present the results of the experience of the call centre approach conducted in BANANGORE sub-district.

Two meetings were organised by the district management team with the support of partners. The aim of the first meeting was to prepare the May 2018 campaign and to extend the call centre approach to the entire BOL district. During the second meeting, several actions were undertaken:

– the responsible sub-districts were informed by the district management team.
– the establishment of a directory of village chiefs
– the validation of progress plans.

**(c) Implementation of vaccination in the island parts of 12 sub-districts**

Before the beginning of vaccination campaign, the managers of sub-districts were asked to provide a list of all villages under their responsibility and the telephone contacts of village, ferry and fishing camp chiefs. The same information was requested simultaneously from the heads of cantons. The coordination team triangulated the information obtained. In the event of a discrepancy between the information provided by managers of subdistricts and that obtained from canton chiefs, to findout the accurate information, the village, ferry or fishing camp chiefs concerned were called on the two telephone numbers obtained to confirm or refute the information obtained. Thus, a telephone directory was established. The telephone numbers of all villages, ferries and fishing camps were recorded in it.

The main coordinator made the telephone call to the village, ferry and fishing camp chiefs on the eve of the launch of vaccination campaign to inform them of the visit of vaccination teams to their respective areas. Verification phone calls were then made to ensure that vaccinators had visited these areas. The lead coordinator, learned some local expressions to ask the following questions: “Hello, have the vaccinators come to your village? Are all the children vaccinated? If the conversation became too long, UNICEF C4D or the driver would take over. Each evening, the information on villages not reached collected by the canton chief was compared with the information collected by the main coordinator and passed on to the health centre managers. A registration code was assigned to each village: “**+**” or “**V**” when the vaccinators had visited the area, “**-**” when the vaccinators had not visited the area, and “**±**” when some children were not vaccinated despite the vaccinators’ passage.

When these telephone calls indicated that vaccinators had not visited the community or that some target children were not being vaccinated, vaccinators were required to return to the communities during the mop-up exercise for effective vaccination. The next day, at the coordination meeting, the senior coordinator provided the district health officer with a list of fully vaccinated, partially vaccinated and unvaccinated villages. The list was based on the team’s progress plan. The district head instructed the relevant health centre managers to send vaccinators back to villages, ferries and fishing camps that had not been reached the previous day.

After the vaccination campaign, partners and the district management team evaluated the campaign. This evaluation focused on the number of villages affected. Afterwards, partners, the district management team, the Head of the sub-districts and EPI managers held feedback meetings, including on activities according to the framework established by the district management team and the call centre activity by partners. Finally, the campaign report was elaborated. The coordination reporter recorded data related to calls made to village, ferry and fishing camp chiefs in the vaccination effectiveness register. After verification and validation by the lead coordinator, these data were entered into the database by a data manager and then verified by a second one.

### Statistical analysis

Word and Excel software were used for word processing and the creation of tables and graphs.

To determine the rate at which villages, ferries, and fishing camps were reached, data from telephone calls made to their chiefs were analysed. The frequency, percentage, and means were calculated to describe the rate of reaching villages, ferries, and fishing camps. The theoretical target of 56.194 is the result of an enumeration survey organized by the Bol district in April 2018. We used Stata 15 software for data analysis. The t-test was used to analyze the change observed before and after the intervention, and the difference between the two periods was compared based on the principle of linear regression.

## Results

The results showed that 87.5% of the 536 heads of villages and communities were alerted (phoned) on the planned vaccinators visit. However, 78.5% of the total communities were vaccinated. Nine percent were not vaccinated. The remaining 12.5% could not be reached due to network problems and they were not vaccinated, throughout the entire vaccination first round. See Table 1. The second round of campaign in May displayed similar trends compared to the first round regarding the localities coverages. See Table 2.

The result showed that the call center had a significantly positive impact on immunization coverage. Compared to the period before the intervention, its increased vaccination coverage by 52% during the first round and 50% during the second round. See Table 3.

Table 4 shows that the use of call centers threw up units that were not visited by the vaccinator and children not vaccinated. It shows that during the April 2018 sub national immunization campaign, 463 children were initially omitted by the vaccinators. However, following the calls made after the initial campaign these children were vaccinated. Similarly, during the fifth round of the main campaign in May 2018 a total of 548 children who were not initially vaccinated but thrown up following the post campaign calls were vaccinated.

**Figure 1:**
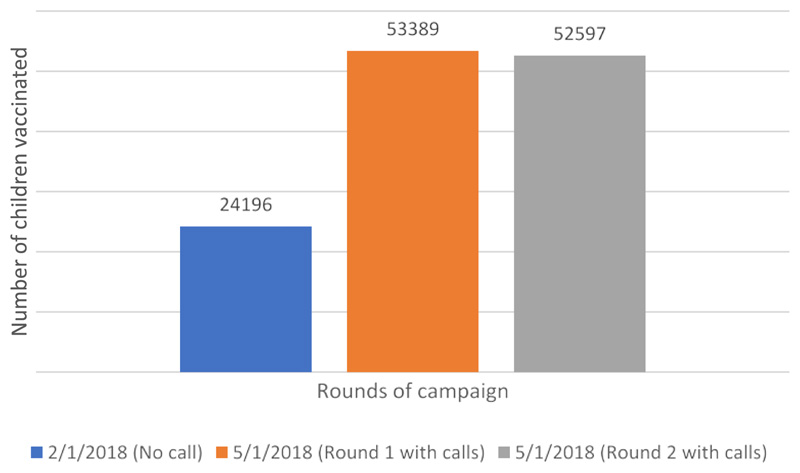


Furthermore, comparative analysis between campaigns using call centers and those without them, showed a remarkable increase in the number of children vaccinated while using the call centers. See Figure 1. With the call centers the number of vaccinated children more than doubled, from 24,196 without call center in February 2018 to 53,389 and 52,597, respectively in the first and second rounds with call centers in May 2018.

## Discussion

Several important results were achieved through the implementation of the call center strategy, that deserves a highlight s on the high value added to the otherwise inefficient routine immunization practices in Chad’s health system. There was a strong citizen and community participation, with 98% of the leaders taking part in the call center approach in the first round, compared to 95% in the second round. This allowed us to have 53,389 children vaccinated in the first round and 52,597 children vaccinated in the second round.

Visits of unconfirmed vaccinators account for about 8% of responses from village chiefs and township chiefs. The non-confirmation is due to non-compliance with the implementation plan by the head of health centres. This could be due to too much workload or poor planning. These planned and unvaccinated villages on the planned days are still caught up the following days during the activity period. Nevertheless, some villages especially difficult to access have been vaccinated after the denunciation of the village chiefs. Thus, more than 1011 children were caught through this approach in 10 villages in the Bol district. The objective is not to prime on board to increase vaccine coverage but to seek to cover all villages. After the vaccination program was given over the phone some village chiefs had to call to confirm the visit of vaccinators without waiting to be called back.

The call center experience during the immunization campaign provided sufficient satisfaction to all involved stakeholders, especially to the partners, the district task force, and especially to the beneficiary community of the intervention. Indeed, Bol District has taken ownership of this successful result and has extended the strategy into routine immunization activities. This brings great value to district’s performance not only in assuring the quality in the implementation of mobile EPI strategies, but also in the authenticity of routine immunization data. In the final analysis, the aim of this report is to share our successful result / outcome with other countries also committed in the polio eradication process.

It is important to note that the same results have been confirmed from other interventions that used the call centers in the delivery of interventions. For instance, the swift control of Ebola virus disease (EVD) outbreak in West Africa was attributed to the engagement of this strategy for early detection of cases, which was later followed by intensive contact tracing efforts and the subsequent isolation of infected secondary cases^9^. On the other hand, delay in detection of cases will speed up the spread of the virus. This is true of the polio eradication. Any unvaccinated child becomes a potential reservoir for the polio virus. This technology provides a fast, simple, and affordable platform for the rapid identification of populations missed during immunization campaign akin to the detection of early- stage disease outbreaks^10^.

In Sierra Leone, toll-free line, Walk-Ins, and community suspects were the three main case notification strategies used in EVD surveillance^9^. Similarly, based on the second global e-Health survey, toll-free telephone services reported 88% for emergency alerts in South-East Asia Region; 63% on lower-middle income Countries and 58% on high income countries. The African Region reported the least (31%) activity in this category. Currently only few African countries had disease-specific emergency toll- free telephone services. One of these is Zimbabwe, which launched a telephone line for citizens to report cholera cases. Madagascar established its own service to support victims of domestic violence while Togo used the emergency toll-free telephone service to obtain information from citizens on H1N1 and TB^9^·^10^.

During the 2012 Ebola outbreak in Uganda, the m-health platform known as mTrac was operationalized. Using a free SMS hotline, communities were engaged in reporting report suspected Ebola cases^11^. Similarly, community members were encouraged to flag any signs of fever and bleeding using the toll-free numbers. The toll-free platform was used in the detection of the first EVD case in Senegal^12^. This technology is thus becoming increasingly deployed for other public health interventions and should be used for immunization services as well.

Among the possible methods or complementary technologies that could be used in the future for the populations located in the area where the network has problems, we can mention the use of “e-tracking” and the involvement of civil society and local non-governmental organizations.

“E-tracking/vaccine tracking system (VTS) is a method of tracking the movement of vaccinators using a telephone application, Mobile Tracker. This application makes it possible to locate a mobile phone and to have a history of its GPS positions. To monitor the progress of the vaccination, the phone with the Mobile Tracker application is given to the vaccinator. The vaccinator is called to work (vaccinate) with the phone on throughout the vaccination. At the end of the vaccination campaign, all these phones are recovered. All you must do is connect them to a computer to read all the vaccinators’ movements^13^.

This method has many drawbacks. It’s a method that allows us to identify the villages reached but does not allow us to know if all the children have been vaccinated. In addition, the vaccinator may forget to turn on the phone or lose it during the vaccination, and despite being charged by the solar power bank, the phone may also discharge, hence the impossibility or interruption of the recording of information.

The involvement of civil society and local nongovernmental organizations appears to be a complimentary method. Members of these associations can supervise the vaccination campaign. Their neutrality makes it possible to have reliable information and, by extension, to increase the rate at which villages are reached^14^.

## Conclusion

After a successful first experience in the Banangorea area of responsibility during the Polio SIAs in April 2018, we extended the strategy throughout Bol district during the first and second rounds of the special SIA in the islands in May 2018. This new approach allowed us to have a telephone directory of the heads of the various entities and to actively involve them in the immunization process. This empowerment of the community doubled the number of children vaccinated. Ownership of this approach by incountry local communities and the different partners by involve in the GPIE Global Polio Eradication Initiative will improve field campaign.

The call center strategy that was implemented in place in this remote region of the country does not pretend to be the panacea for the best strategies that can sustainably improve immunization coverage. We must continue to draw and share lessons learned with national and local officials to improve / potentiate the chances of success of such unique and future and initiatives. While this is an inexpensive and easily achievable strategy, it remains limited in several ways, as it requires the continued mobilization of the ongoing commitment of local leaders and community leaders in support of the community health officials for adoption and transferability to other settings in the similar context of the country and elsewhere.

## Figures and Tables

**Table 1:** Results of the first round of the immunization campaign and Use of Call Centers in May 2018 (% in parentheses)

Vaccination day	# village heads contacted & villages vaccinated	# village heads contacted, but villages not vaccinated	# village heads not contacted	Total
D1	119 (74.4)	21 (13.1)	20 (12.5)	160
D2	199 (87.3)	10 (4.4)	19 (8.3)	228
D3	76 (67.3)	17 (15.0)	20 (17.7)	113
D4	27 (77.1)	0 (0.0)	8 (22.9)	35
Total	**421(78.5)**	**48 (9.0)**	**67 (12.5)**	**536**

**Table 2:** Results of the first round of the immunization campaign and Use of Call Centers in the second round in May 2018 (% in parentheses)

Day	Village head contacted & number and entities were vaccinated	Village head contacted, and entities were not vaccinated	Uncontacable village head	Total
D1	174 (78.7)	5 (2.3)	42 (19.0)	221
D2	124 (75.1)	13 (7.9)	28 (17.0)	165
D3	94 (79.0)	11 (9.2)	14 (11.8)	119
D4	26 (55.3)	0 (0.0)	21 (44.7)	47
Total	**418 (75.7)**	**29 (5.3)**	**105 (19.0)**	**552**

**Table 3:** Call center effect on vaccination coverage n (56194)

Variables		Before intervention (%)	After intervention (%)	Attributable change (CI 95%)	p
Immunization coverage					
	Round 1 with Call	**(43.0%)**	**(95.0%)**	+0.52 (0.51 to 0.53)	**<0,001** ^t^
Immunization coverage					
	Round 2 with Call	**(43.0%)**	**(94.0%)**	+0.51 (0.50 to 0.52)	**<0,001** ^t^

With n=number of subjects

p=p-value

and^t^ =t test

**Table 4:** Summary of the calls made during the Island activities

	Districts	SNID April 2018						Island vaccination Rounds 1,2,3,4						5th Round					
		# of call made	# of visits confirmed	# of visits not confirmed	# of unreachable phone numbers	# of villages not vaccinated discovered after calls	# of children caught after calls	# of call made	# of visits confirmed	# of visits not confirmed	# of unreachable phone numbers	# of villages not vaccinated discovered after calls	# of children caught after calls	# of call made	# of visits confirmed	# of visits not confirmed	# of unreachable phone numbers	# of villages not vaccinated discovered after calls	# of children caught after calls
1	Bol	44	41	3	0	3	463	1088	839	77	172	0	0	307	294	0	13	0	0
2	Baga Sola													87	80	7	0	7	548
3	Karal													76	63	13	59	0	0
**TOTAL**		**44**	**41**	**3**	**0**	**3**	**463**	**1088**	**839**	**77**	**172**	**0**	**0**	**470**	**437**	**20**	**72**	**7**	**548**
